# Supplementary material for: An automated software-assisted approach for exploring metabolic susceptibility and degradation products in macromolecules using high-resolution mass spectrometry
Source: PLoS One. 2025 Aug 13;20(8):e0324668. doi: 10.1371/journal.pone.0324668 (PMC12349704; doi:10.1371/journal.pone.0324668)
Supplement: S1 File — (PDF) [file pone.0324668.s001.pdf]

S1. Deslorelin – MAM Settings

S2. Deslorelin – MIM Settings

S3. Goserelin – MAM Settings

S4. Goserelin – MIM Settings

S5. Buserelin – MAM Settings

S6. Buserelin – MIM Settings

S7. Histrelin – MAM Settings

S8. Histrelin – MIM Settings

S9. Leuprolide – MAM Settings

S10. Leuprolide – MIM Settings

S11. Secretin – MAM Settings

S12. Secretin – MIM Settings

S13. Octreotide – MAM Settings

S14. Octreotide – MIM Settings

S15. Oxytocin – MAM Settings

S16. Oxytocin – MIM Settings

S17. Calcitonin – MAM Settings

S18. Calcitonin – MIM Settings

S19. GLP-1 – MAM Settings – DPP-4

S20. GLP-1 – MIM Settings – DPP-4

S21. Liraglutide – MAM Settings – DPP-4

S22. Liraglutide – MIM Settings – DPP-4

S23. Taspoglutide – MAM Settings – DPP-4

S24. Taspoglutide – MIM Settings – DPP-4

S25. Semaglutide - MAM Settings

S26. Semaglutide - MIM Settings

S27. Exenatide – MAM Settings – DPP-4

S28. Exenatide – MIM Settings – DPP-4

S29. GLP1 – MAM Settings – NEP

S30. GLP1 – MIM Settings – NEP

S31. Liraglutide – MAM Settings – NEP

S32. Liraglutide – MIM Settings – NEP

S33. Taspoglutide – MAM Settings – NEP

S34. Taspoglutide – MIM Settings – NEP

S35. Somatostatin – MAM Settings – DDA

S36. Somatostatin – MIM Settings – DDA

S37. Analogue 6 – MAM Settings – DDA

S38. Analogue 6 – MIM Settings – DDA

S39. Analogue 30 – MAM Settings – DDA

S40. Analogue 30 – MIM Settings – DDA

S41. Analogue 31 – MAM Settings – DDA

S42. Analogue 31 – MIM Settings – DDA

S43. Analogue 35 – MAM Settings – DDA

S44. Analogue 35 – MIM Settings – DDA

S45. Analogue 64 – MAM Settings – DDA

S46. Analogue 64 – MIM Settings – DDA

S47. Analogue 65 – MAM Settings – DDA

S48. Analogue 65 – MIM Settings – DDA

S49. Analogue 95 – MAM Settings – DDA

S50. Analogue 95 – MIM Settings - DDA

S51. Somatostatin – MAM Settings – DIA

S52. Somatostatin – MIM Settings – DIA

S53. Analogue 6 – MAM Settings – DIA

S54. Analogue 6 – MIM Settings – DIA

S55. Analogue 30 – MAM Settings – DIA

S56. Analogue 30 – MIM Settings – DIA

S57. Analogue 31 – MAM Settings – DIA

S58. Analogue 31 – MIM Settings – DIA

S59. Analogue 35 – MAM Settings – DIA

S60. Analogue 35 – MIM Settings – DIA

S61. Analogue 64 – MAM Settings – DIA

S62. Analogue 64 – MIM Settings – DIA

S63. Analogue 65 – MAM Settings – DIA

S64. Analogue 65 – MIM Settings – DIA

S65. Analogue 95 – MAM Settings – DIA

S66. Analogue 95 – MIM Settings – DIA

S67. Antisense Oligonucleotide – MAM Settings – Not Expanded

S68. Antisense Oligonucleotide – MAM Settings – Expanded

S69. Antisense Oligonucleotide – MIM Settings – Not Expanded

S70. BIO-1211 – MAM Settings – CatG

S71. BIO-1211 – MAM Settings – MMP12

S72. BIO-1211 – MAM Settings – NE

S73. BIO-1211 – MAM Settings – Trypsin

S74. CSP7 – MAM Settings – CatG

S75. CSP7 – MAM Settings – MMP12

S76. CSP7 – MAM Settings – NE

S77. CSP7 – MAM Settings – Trypsin

S78. Peptide T – MAM Settings – CatG

S79. Peptide T – MAM Settings – MMP12

S80. Peptide T – MAM Settings – NE

S81. Peptide T - MAM Settings – Trypsin

S82. BIO-11006 - MAM Settings – CatG

S83. BIO-11006 - MAM Settings – MMP12

S84. BIO-11006 - MAM Settings – NE

S85. BIO-11006 - MAM Settings – Trypsin

S86. SPX-101 – MAM Settings – CatG

S87. SPX-101 – MAM Settings – MMP12

S88. SPX-101 – MAM Settings - NE

S89. SPX-101 – MAM Settings – Trypsin

S90. M10 Peptide - MAM Settings – CatG

S91. M10 Peptide - MAM Settings – MMP12

S92. M10 Peptide - MAM Settings - NE

S93. M10 Peptide - MAM Settings – Trypsin

S94. Gonadorelin - MAM Settings – CatG

S95. Gonadorelin - MAM Settings – MMP12

S96. Gonadorelin - MAM Settings - NE

S97. Gonadorelin – MAM Settings – Trypsin

S98. Leuprolide - MAM Settings – MMP12

S99. Leuprolide - MAM Settings – NE

S100. Leuprolide - MAM Settings – Trypsin

S101. Deslorelin - MAM Settings – CatG

S102. Deslorelin - MAM Settings – MMP12

S103. Deslorelin - MAM Settings – NE

S104. Deslorelin – MAM Settings – Trypsin

S105. Triptorelin - MAM Settings – CatG

S106. Triptorelin - MAM Settings – MMP12

S107. Triptorelin - MAM Settings – NE

S108. Triptorelin - MAM Settings – Trypsin

S109. NAS-911 - MAM Settings – CatG

S110. NAS-911 - MAM Settings – MMP12

S111. NAS-911 - MAM Settings – NE

S112. NAS-911 - MAM Settings – Trypsin

S113. LDTRYLEQLHKLY - MAM Settings – CatG

S114. LDTRYLEQLHKLY - MAM Settings – MMP12

S115. LDTRYLEQLHKLY - MAM Settings – NE

S116. MMI-0100 - MAM Settings – CatG

S117. MMI-0100 - MAM Settings – MMP12

S118. MMI-0100 – MAM Settings – NE

S119. Salmon Calcitonin – MAM Settings – CatG

S120. Salmon Calcitonin – MAM Settings – MMP12

S121. Salmon Calcitonin – MAM Settings – NE

S122. Salmon Calcitonin – MAM Settings – Trypsin

S123. Carbetocin – MAM Settings – CatG

S124. Carbetocin – MAM Settings – MMP12

S125. Carbetocin – MAM Settings – NE

S126. Carbetocin – MAM Settings – Trypsin

S127. Atosiban - MAM Settings – MMP12

S128. Atosiban - MAM Settings – NE

S129. Atosiban - MAM Settings – Trypsin

S130. Octreotide - MAM Settings – CatG

S131. Octreotide - MAM Settings – MMP12

S132. Octreotide - MAM Settings – NE

S133. Octreotide - MAM Settings – Trypsin

S134. Felypressin – MAM Settings – CatG

S135. Felypressin – MAM Settings – MMP12

S136. Felypressin – MAM Settings – NE

S137. Lypressin – MAM Settings – CatG

S138. Lypressin – MAM Settings – MMP12

S139. Lypressin – MAM Settings – NE

S140. Desmopressin – MAM Settings – CatG

S141. Desmopressin – MAM Settings – MMP12

S142. Desmopressin – MAM Settings – NE

S143. Desmopressin – MAM Settings – Trypsin

S144. Vasopressin - MAM Settings – CatG

S145. Vasopressin - MAM Settings – MMP12

S146. Vasopressin - MAM Settings – NE

S147. Lanreotide - MAM Settings – MMP12

S148. Lanreotide - MAM Settings – NE

S149. Lanreotide - MAM Settings – Trypsin

S150. Vapreotide – MAM Settings – CatG

S151. Vapreotide - MAM Settings – MMP12

S152. Vapreotide - MAM Settings – NE

S153. Vapreotide - MAM Settings – Trypsin

S154. Somatostatin - MAM Settings – CatG

S155. Somatostatin - MAM Settings – MMP12

S156. Somatostatin - MAM Settings – NE

S157. Somatostatin - MAM Settings – Trypsin

S158. Iseganan - MAM Settings – CatG

S159. Iseganan - MAM Settings – MMP12

S160. Iseganan - MAM Settings – NE

S161. Human Insulin – MAM Settings

S162. Human Insulin – MIM Settings
