# Supplementary material for: An automated software-assisted approach for exploring metabolic susceptibility and degradation products in macromolecules using high-resolution mass spectrometry
Source: PLoS One. 2025 Aug 13;20(8):e0324668. doi: 10.1371/journal.pone.0324668 (PMC12349704; doi:10.1371/journal.pone.0324668)
Supplement: S13 File — (PDF) [file pone.0324668.s013.pdf]

MassMetaSite Experiment Settings-1.0.0

External LCMS File converter enabled=true

Negative adduct, Cl-=false

GSH fragment ion extras z=2=76.0393

Neutral adduct, Acetonitrile=false

Mass model selected=MAM

Reactions group|#3#Chemistry==0

Radio peak detection smoothing=HIGH

Include neutral losses=false

Filter fragments above ppm value=0

Minimum mass=100

negative control area ratio value=1

Bond breaking reorganization enabled, even electron, MS/MS=true

Bond breaking reorganization enabled, even electron, MS=true

Mass score filter enabled=false

GSH fragment ion extras=130.0499;162.0219;177.0328;179.0485

Chromatogram automatic filtering threshold=0.97

UV peak area enabled=false

Preset UV Wavelength enabled=false

Sum area, Positive Adduct area=false

Split computed DRM peaks=true

Bond breaking reorganization enabled, Aryl Methoxy, MS=false

Bond breaking reorganization enabled, Benzyl alcohol, MS/MS=false

Min retention time range=0

Is macromolecule mode=true

Internal standard retention time=2.26

CYP(s)=LIVER

Positive adduct, K+=false

Negative control file signal filtering threshold=0

Sum area, Dimer area=false

Filter unexpected metabolites using retention time=true

GSH neutral loss extras z=2=

Number of metabolite generations=2

GSH neutral loss triggers=129.0426

metabolize from termini only=false

Bond breaking reorganization enabled, Benzyl alcohol, MS=false

MS/MS automatic filtering threshold=0.98

Import Mode=ONLY2D

Signal filtering=THRESHOLD

Bond breaking reorganization enabled, odd electron, MS/MS=true

Isotope Labels=

Positive adduct, NH4+=false

Bond breaking reorganization enabled, Amide water loss, MS/MS=false

Bond breaking reorganization enabled, Aryl Methoxy, MS/MS=false

Split modified metabolites=false

Activate GSH mode=false

Ionization Mode=[M+H]+

Bond breaking reorganization enabled, odd electron, MS=true

UV/MS time delay tolerance=0|0

Mass relative score filter enabled=false

Peak area threshold=1.0E-4

Scan filtering=AUTO

Split unexpected metabolites=false

Metabolite bond breaking limit=1

Radio peak area enabled=false

Internal standard UV wavelength=200

Isotopic labeling mode=Peptide

Sum area, Negative Adduct area=false

Ignore metabolites stereochemistry enabled=true

Negative adduct, Br-=false

Peak detection smoothing=HIGH

Include multiple charges=true

Bond breaking reorganization enabled, Alkyl Sulphate, MS=false

Bond breaking reorganization enabled, N-oxide, MS=true

Maximum number of intermonomer reactions=2

Monomer bond definitions rules=amide bond

Bond breaking reorganization enabled, electron dissociation, MS/MS=false

Isotopic labeling enabled=false

Negative adduct, Acetate=false

Mass score threshold=0

Negative adduct, Formate=false

Sum chromatogram signals enabled=false

Bond breaking reorganization enabled, electron dissociation, MS=false

Same peak tolerance unit=amu

Max metabolite count enabled=false

Expand unknowns monomers=false

Automatic structure selection=true

Consensus Model=true

Internal standard m/z=556.267

Isotope filter enabled=false

Sum area, Neutral Loss area=false

Mass relative score threshold=0

Neutral adduct, Methanol=false

GSH neutral loss extras=75.0320;146.0692;232.0696;249.0961;273.0961;275.1117;305.0682;307.0838

UV Wavelength selection method=WAVELENGTH_SEL_RATIO

Rescue computed DRM peaks enabled=true

Internal standard name=IS

Error tolerance for isotope label ratio=0

Sum area, Neutral Adduct area=false

Include multimers=false

Fluo peak area enabled=false

Filter metabolites by ppm=true

Positive adduct, Na+=false

Max retention time range=0

Isotope label abundances=0

Collapse MS/MS levels=false

Filter unexpected metabolites using MSMS=true

Theoretical isotope ratio threshold=0

Break metabolite enabled=true

Bond breaking reorganization enabled, Amide water loss, MS=false

Reactions group|#1#Peptide=Amide Hydrolysis | Disulfide reduction=1

Maximum MS/MS level=2

Bond breaking reorganization enabled, N-oxide, MS/MS=false

GSH fragment ion triggers z=2=130.0499

Maximum charge state=6

Acquisition scan filtering threshold=120

GSH neutral loss triggers z=2=

Absolute peak area threshold=0

Isotope cluster filtering=0

Saturation threshold=0

Max metabolite limit=20

GSH fragment ion triggers=308.0911

Fluo peak area threshold=0.002

Negative adduct, TFA=false

Ignore redundant metabolites enabled=true

Bond breaking reorganization enabled, Alkyl Sulphate, MS/MS=false

Bond breaking limit=2

Activate Peptide mode=true

Isotope filter tolerance=0.2

m/z tolerance for isotope detection=1

Fragment only b/y bond=false

Resolving power for isotope detection=10000

Charge deconvolution=false

Internal standard retention time tolerance=0.1

Radio peak area threshold=0.002

Same peak tolerance=0.025

Retention time range enabled=false

Filter metabolites without formula=false

Break 6-membered heteroaromatic rings=false

Filter fragments by ppm=false

lock mass=0

lock mass tolerance=0

Oxidation during MS/MS=false

Preset UV Wavelength=220

Include unexpected metabolites=true

Ignore redundant metabolites fraction=30

use negative control area ratio=false

Filter metabolites above ppm value=10

Computation mode=TEC

MS automatic filtering threshold=0.98

Reactions group|#2#Oligo=Phosphoester Hydrolysis=1

Mass Spectrometer=Waters Q-TOF

UV/MS time delay=NO

UV peak area threshold=0.002

Fluo peak detection smoothing=3

Execution timeout=60

Merge MSMS from multiple charge states=false
