# Supplementary material for: An automated software-assisted approach for exploring metabolic susceptibility and degradation products in macromolecules using high-resolution mass spectrometry
Source: PLoS One. 2025 Aug 13;20(8):e0324668. doi: 10.1371/journal.pone.0324668 (PMC12349704; doi:10.1371/journal.pone.0324668)
Supplement: Attachment — Submitted filename: Response to Reviewers.docx [file pone.0324668.s016.docx]

Dear Editor and Reviewers,

We sincerely thank you for your thoughtful and constructive comments on our manuscript. We have carefully considered each point raised and have revised the manuscript accordingly to address all concerns. Below, we provide detailed responses to the academic editor and reviewers’ comments along with explanations of the changes made.

We appreciate the opportunity to improve our work and believe that these revisions have significantly strengthened the manuscript.

Thank you for your time and consideration.

**RESPONSE TO ACADEMIC EDITOR**

**1. Please ensure that your manuscript meets PLOS ONE's style requirements, including those for file naming. The PLOS ONE style templates can be found at https://journals.plos.org/plosone/s/file?id=wjVg/PLOSOne_formatting_sample_main_body.pdf and https://journals.plos.org/plosone/s/file?id=ba62/PLOSOne_formatting_sample_title_authors_affiliations.pdf**

The revised manuscript now adheres to PLOS ONE’s style requirements, including those related to file naming.

**2. Please note that PLOS ONE has specific guidelines on code sharing for submissions in which author-generated code underpins the findings in the manuscript. In these cases, we expect all author-generated code to be made available without restrictions upon publication of the work. Please review our guidelines at https://journals.plos.org/plosone/s/materials-and-software-sharing#loc-sharing-code and ensure that your code is shared in a way that follows best practice and facilitates reproducibility and reuse.**

The software used in my manuscript is a proprietary tool developed and maintained by a third party. Due to licensing restrictions, the source code itself cannot be distributed directly. However, the developers provide access via a publicly available AWS-hosted server that includes a free trial option. This allows readers to use the software and reproduce the analyses as described in the manuscript. The link to access to the AWS-hosted server: https://oniro.mass-analytica.com/

**3. Thank you for stating in your Funding Statement: [This work has been partially supported by Doctorats Industrials, AGAUR, Generalitat de Catalunya. Industrial Doctorate grant 00002/2023.].**

**Please provide an amended statement that declares *all* the funding or sources of support (whether external or internal to your organization) received during this study, as detailed online in our guide for authors at http://journals.plos.org/plosone/s/submit-now. Please also include the statement “There was no additional external funding received for this study.” in your updated Funding Statement.**

**Please include your amended Funding Statement within your cover letter. We will change the online submission form on your behalf.**

This work has been partially supported by Doctorats Industrials, AGAUR, Generalitat de Catalunya. Industrial Doctorate grant 00002/2023, that funded all travel expenses related to this research. There was no additional external funding received for this study.

The Funding Statement has been included in the cover letter as requested.

**4. Thank you for stating the following in the Competing Interests section: [The author declare the following competing financial interest(s): P.C. is an employee of Lead Molecular Design, S.L., and I.Z. is the CEO of the company. Lead Molecular Design, S.L. develops analytical software, including MassMetaSite and Oniro, which were used in this study.].**

**We note that you received funding from a commercial source: [Lead Molecular Design]**

**Please provide an amended Competing Interests Statement that explicitly states this commercial funder, along with any other relevant declarations relating to employment, consultancy, patents, products in development, marketed products, etc.**

**Within this Competing Interests Statement, please confirm that this does not alter your adherence to all PLOS ONE policies on sharing data and materials by including the following statement: "This does not alter our adherence to PLOS ONE policies on sharing data and materials.” (as detailed online in our guide for authors http://journals.plos.org/plosone/s/competing-interests). If there are restrictions on sharing of data and/or materials, please state these. Please note that we cannot proceed with consideration of your article until this information has been declared. Please include your amended Competing Interests Statement within your cover letter. We will change the online submission form on your behalf.**

P.C., F.F, and A.G are employees of Lead Molecular Design, S.L., and I.Z. is the CEO of the company. Lead Molecular Design, S.L. develops analytical software, including MassMetaSite and Oniro, which were used in this study. L.C is an employee for Mass Spec Analytica, S.L, the software distributor. Due to licensing restrictions, the source code of Oniro cannot be distributed directly. However, the software is accessible via a publicly available AWS-hosted server that includes a free trial option, allowing readers to use the software and reproduce the analyses described in the manuscript (<https://oniro.mass-analytica.com/>). This does not alter our adherence to PLOS ONE policies on sharing data and materials.

**5. Thank you for stating the following in the Acknowledgments Section of your manuscript: [This work was supported by the Generalitat de Catalunya and Lead Molecular Design S.L through the Industrial Doctorate grant 00002/2023.]**

**We note that you have provided funding information that is not currently declared in your Funding Statement. However, funding information should not appear in the Acknowledgments section or other areas of your manuscript. We will only publish funding information present in the Funding Statement section of the online submission form.**

**Please remove any funding-related text from the manuscript and let us know how you would like to update your Funding Statement. Currently, your Funding Statement reads as follows: [This work has been partially supported by Doctorats Industrials, AGAUR, Generalitat de Catalunya. Industrial Doctorate grant 00002/2023.].**

**Please include your amended statements within your cover letter; we will change the online submission form on your behalf.**

The funding information has been removed from the Acknowledgements section of the revised manuscript and has been included in the cover letter.

Please update the Funding Statement as: This work has been partially supported by Doctorats Industrials, AGAUR, Generalitat de Catalunya. Industrial Doctorate grant 00002/2023, that funded all travel expenses related to this research. There was no additional external funding received for this study.

**RESPONSE TO REVIEWERS**

**REVIEWER #1:**

**1. The authors have provided previous work citations for their software [6,7], which suggests some automating data analysis already exists. It would strengthen the paper highlight the novelty of this work such as is it the integration of peak detection algorithms for macromolecules (monoisotopic vs most abundant), the visualization approach.**

This point has been addressed in the revised manuscript through a rewriting of the Introduction section. The novelty of the work is now clearly highlighted (see lines 90-105), including the introduction of a new peak detection algorithm based on the most abundant mass (MaM) peak, which is compared to the monoisotopic mass (MiM) approach traditionally used for small molecules. Additionally, the revised manuscript emphasizes three distinct visualization strategies for macromolecules—expanded, non-expanded, and hybrid modes—and discusses their impact on computational efficiency and the identification of biotransformation sites in macromolecules.

**2. Authors mentioned two peak detection techniques (MiM vs. MaM) and using fragment match scoring. For big molecules, for instance, how were mass inaccuracies (ppm) managed? Were several charge states taken into account and deconvoluted?**

The management of mass inaccuracies (ppm) and the handling of multiple charge states have been carefully considered in our methodology. Mass accuracy is controlled through user-defined mass tolerance settings, which are detailed in the supplementary information. This tolerance is applied by comparing the theoretical m/z values of predicted metabolite structures with the observed m/z values in the experimental spectra. The metabolite structures are generated virtually by applying specific metabolic transformations to the parent compound. For example, in the case of amide hydrolysis, the transformation corresponds to the addition of an OH group to the carboxylic terminus and a hydrogen atom to the amine terminus. These biotransformations are selectable in the software settings.

In addition to considering the singly protonated species, multiple charge states are incorporated into the analysis. For each metabolite structure, multiple potential m/z values are computed corresponding to different charge states, which can also be specified by the user (supplementary material). The algorithm clusters all ions in the full scan chromatogram that share the same retention time peak, effectively grouping all charge states corresponding to a single chemical species. Consequently, there is no requirement for deconvolution since all relevant charge states are inherently considered during the process.

This approach is analogous to that commonly used for small molecule analysis and has been described extensively in prior studies [1, 2, 3, 4, 5]; thus, further elaboration is beyond the scope of this manuscript. Specifically, for the monoisotopic mass (MiM) peak detection, the theoretical m/z used for comparison corresponds to the monoisotopic mass of the metabolite. In contrast, for the most abundant mass (MaM) approach, the m/z corresponds to the isotope with the highest natural abundance intensity, calculated based on the isotopic distribution derived from the formula of the compound.

(1) *Zamora I, Fontaine F, Serra B, Plasencia G. High-throughput, computer assisted, specific MetID. A revolution for drug discovery. Drug Discov Today Technol. 2013 Spring;10(1):e199-205. doi: 10.1016/j.ddtec.2012.10.015. PMID: 24050248.*
(2) *Ahlqvist M, Leandersson C, Hayes MA, Zamora I, Thompson RA. Software-aided structural elucidation in drug discovery. Rapid Commun Mass Spectrom. 2015 Nov 15;29(21):2083-9. doi: 10.1002/rcm.7364. PMID: 26443410.*
(3) *Li AC, Chovan JP, Yu E, Zamora I. Update on hydrocodone metabolites in rats and dogs aided with a semi-automatic software for metabolite identification Mass-MetaSite. Xenobiotica. 2013 Apr;43(4):390-8. doi: 10.3109/00498254.2012.715697. Epub 2012 Aug 30. PMID: 22931213.*
(4) *Zelesky V, Schneider R, Janiszewski J, Zamora I, Ferguson J, Troutman M. Software automation tools for increased throughput metabolic soft-spot identification in early drug discovery. Bioanalysis. 2013 May;5(10):1165-79. doi: 10.4155/bio.13.89. PMID: 23721441.*
(5) *Bonn, B., Leandersson, C., Fontaine, F. and Zamora, I. (2010), Enhanced metabolite identification with MS^E^ and a semi-automated software for structural elucidation. Rapid Commun. Mass Spectrom., 24: 3127-3138. https://doi.org/10.1002/rcm.4753*

**3. The authors claim “substantial reductions in processing time” and “consistent identification of degradation products” compared to prior work, but these claims need quantitative support. For processing time, it would be helpful to present a table or bar chart showing the workflow’s runtime for each dataset and algorithm (MiM vs MaM), alongside the runtimes of previous methods (if available from literature or the authors’ earlier pipelines). The statement "processing time ranged from 5 minutes to 2 hours per experiment," for instance, is helpful, but it would be more better to show how the specific circumstances (eg, large pepitde took 2h) and the extent of the improvement. Likewise, does "consistent identification" imply that the same metabolites were discovered in each replicate and also does the result of degradation matches previous reports?**

We have revised the manuscript to provide quantitative support for our claims regarding processing time by adding a new Table 10 and explanation (see lines 675 – 685, under the new section titled Processing time in the Results and discussion). Processing time is primarily influenced by the size and molecular weight of the peptide, which is why comparisons have been made specifically between the two visualization modes (non-expanded vs. expanded). The observed differences are due to the fragmentation strategies that use these algorithms: the non-expanded mode performs fragmentation at the monomer level, using predefined ion types (e.g., a, b, c, x, y, z ions), whereas the expanded mode simulates fragmentation at the atomic level by disconnecting all chemical bonds. This results in a significantly larger number of theoretical fragments, thereby increasing processing time. Additionally, the statement "processing time ranged from 5 minutes to 2 hours per experiment" has been clarified in the revised manuscript (see lines 43-44).

Regarding the claim of consistent identification, this refers to the reproducible identification of the same metabolites and structural assignments in agreement with previously published studies. Specifically, the identified metabolites in the following datasets were compared against prior literature:

- Dataset 1 and 2 results were benchmarked against: Radchenko et al., *PLOS ONE*, 2018 (<https://doi.org/10.1371/journal.pone.0200772>)
- Dataset 3 was compared to: Radchenko et al., PhD Thesis, 2018 ([https://www.tdx.cat/handle/10803/665008](https://www.tdx.cat/handle/10803/665008))
- Dataset 5 was compared to: Wesche et al., *J Pharm Biomed Anal*, 2022 (<https://doi.org/10.1016/j.jpba.2021.114518>)
- Dataset 6 was compared to: Manolopoulou et al., *J Biol Chem*, 2009 (<https://doi.org/10.1074/jbc.M900068200>)

**4. Does these new algorithms and workflow are available to other researchers. It will better to mentioned whether the software is open-source or can be accessed (and how).**

The software described in this work is a commercial product and is not open-source. However, a publicly accessible instance is available online with example datasets ([https://oniro.mass-analytica.com/](https://oniro.mass-analytica.com/)), allowing users to explore the functionality and evaluate the new algorithms and workflow. Additionally, a free two-month evaluation license can be requested, enabling full installation and use of the software during that period. Requests for evaluation access can be submitted through the commercial distributor at [www.mass-analytica.com](http://www.mass-analytica.com).

**5. Authors are suggested to go for full experimental validation (e.g. NMR or orthogonal MS for each metabolite), to support strong claims in the Conclusion and Abstract (“accurate prediction of metabolite structures”, “proven the analysis”, “greater confidence”).**

We fully acknowledge the importance of experimental validation, such as NMR or orthogonal MS, especially in the context of definitive structure confirmation. However, the scope of this study is specifically focused on metabolite identification during the drug discovery phase, where compounds are often available only in small quantities and not subject to purification. Under these conditions, full structural elucidation by techniques like NMR is generally not feasible and falls outside the intended use case of the methodology presented.

The aim of our work is to demonstrate an automated approach for structure elucidation based on high-resolution MS/MS data, which is particularly relevant during early screening when rapid and informative metabolic characterization is required. In the development phase, where larger quantities of material are available, we agree that full synthesis and biological/toxicological profiling of metabolites is essential. However, that is not the focus of the current paper.

To ensure confidence in our results, we compared the identified metabolites to those reported in previously published studies wherever possible. Specifically:

- Dataset 1 and 2 results were benchmarked against: Radchenko et al., *PLOS ONE*, 2018 ([https://doi.org/10.1371/journal.pone.0200772](https://doi.org/10.1371/journal.pone.0200772))
- Dataset 3 was compared to: Radchenko et al., PhD Thesis, 2018 ([https://www.tdx.cat/handle/10803/665008](https://www.tdx.cat/handle/10803/665008))
- Dataset 5 was compared to: Wesche et al., *J Pharm Biomed Anal*, 2022 (<https://doi.org/10.1016/j.jpba.2021.114518>)
- Dataset 6 was compared to: Manolopoulou et al., *J Biol Chem*, 2009 (<https://doi.org/10.1074/jbc.M900068200>)

In cases where no published references were available, the proposed structures were the only ones compatible with the observed m/z values and fragmentation patterns obtained. We have clarified the scope of the paper to the discovery space.

**Minor Comments**

**1. Ensure consistency in heading capitalization. eg. “Materials and methods” and “Results and Discussion”. Authors are advised to follow the journal’s style and apply it throughout the manuscript.**

This point has been addressed in the revised manuscript.

**2. In Tables, either replace “Nº of dataset” with “Dataset number” or simply “Dataset”. Avoid using “Nº” (the degree symbol) in formal tables or define the same in its caption.**

This point has been addressed in the revised manuscript.

**3. Both the Abstract and Conclusion summarize similar findings, improve both.**

This point has been addressed in the revised manuscript.

**4. For consistency, either write out “minutes” or “min” uniformly, authors have used both which is need to be improved.**

This point has been addressed in the revised manuscript.

**5. Check for grammar and acronyms at first use.**

This point has been addressed in the revised manuscript.

**REVIEWER #2:**

**Major comments:**

**1. Please rewrite the introduction, including citations, that provide a scientific basis for the claims made compared to the existing literature.**

This point has been addressed in the revised manuscript.

**2. In the introduction section, authors should:**

**(a) briefly explain how metabolites contribute to therapeutic efficacy, toxicity, and drug-drug interactions.**

This point has been addressed in the revised manuscript through a rewriting of the Introduction section. It now clearly explains the potential adverse effects of metabolites, including toxicity and chemical reactivity that may interfere with the metabolism of co-administered drugs and increase the risk of drug-drug interactions, see lines 56-58.

**(b) highlight the importance of metabolite profiling that leads to the development of an effective drug in the introduction.**

This point has been addressed in the revised manuscript through a rewriting of the Introduction section. Lines 59-63 now emphasize the role of MetID in the development of effective therapeutic agents.

**(c) explain the adverse effects of metabolites, preventing adverse drug reactions, and guiding clinical efforts.**

This point has been addressed in the revised manuscript through a rewriting of the Introduction section. It now highlights how metabolite identification supports the prevention of adverse drug reactions and informs clinical monitoring strategies, ultimately guiding personalized medicine efforts.

**(d) expand tools for in silico models and in vitro assays, identifying drug metabolites. Advantages and limitations of these approaches regarding the present study.**

The Introduction section has been rewritten in the updated manuscript, and the paragraph referencing the sentence in question is no longer included. While the current version does not explicitly discuss the advantages and limitations of *in silico* models and *in vitro* assays, the focus has been refined to highlight the specific challenges and solutions related to metabolite identification of macromolecules using LC-HRMS data. We believe this provides a more targeted context for the scope and objectives of the study.

**(e) detail more clear explanation of hybrid approaches with an example. What specific features of these software solutions faster than existing tools in terms of capabilities and performance?**

As in the previous case, the Introduction section has been rewritten in the updated manuscript, and the paragraph originally referring to hybrid approaches is no longer included. However, the main challenges associated with automated metabolite identification of macromolecules are now briefly discussed in lines 66-72 of the revised manuscript.

Additionally, the specific improvements introduced by the new approaches implemented in the software are clarified throughout the *Results and discussion* section. These enhancements include faster data processing, improved interpretability of biotransformations for macromolecules, and a peak detection algorithm tailored for MetID of macromolecules, which provides greater confidence in structural predictions and reduces the incidence of false positives.

**(f) Please highlight any scientific report citing unsuitability for the conventional approach for larger and intricate molecules such as peptide oligonucleotide and antibodies, with increasing challenges with the increase in size.**

This point has been addressed in the revised manuscript (see lines 73-77) through a rewriting of the Introduction section. Specifically, we have cited a scientific report^1^ that highlights the exponential growth in the number of theoretical fragments and possible metabolite structures as the molecular size increases, particularly in macromolecules. This complexity, driven by the growing number of atoms, makes conventional small-molecule metabolite identification approaches unsuitable for larger molecules.

To further clarify this limitation, conventional small-molecule MetID tools require extensive memory and computation time when applied to macromolecules, often rendering the analysis unmanageable within practical time frames. In contrast, our approach introduces a monomer-based representation strategy that significantly reduces computational complexity. By treating macromolecules as a sequence of monomers, the number of fragments is limited to a defined set (e.g., a, b, c, x, y, z ions in peptide fragmentation), and the number of metabolite structures is reduced by applying biotransformations at the monomer level. Instead of generating atomic-level chemical modifications, our method calculates the transformation as a change in monomer formula from the parent to the hypothetical metabolite, which drastically reduces the number of virtual metabolite structures required.

This monomer-based strategy, however, does introduce trade-offs. It may limit detailed structural resolution at the atomic level and restrict the chemical structure information available for downstream analyses such as chemical similarity searches. To address this, we also support a hybrid representation approach where part of the molecule is treated using monomer representation—offering improved computational efficiency—while another part is processed at the atom/bond level, providing enhanced structural detail where needed. This hybrid approach allows users to prioritize structural accuracy or processing efficiency depending on the metabolic relevance of different molecular regions.

*(1) Cuyckens F, Dillen L, Cools W, Bockx M, Vereyken L, de Vries R, Mortishire-Smith RJ. Identifying metabolite ions of peptide drugs in the presence of an in vivo matrix background. Bioanalysis. 2012;4(5):595–604. doi: 10.4155/bio.11.333 PMID: 22409556*

**(g) Line 99:101 – The article aims to address the challenges in the processing of only macromolecules, or macromolecules and small molecules, as described in the manuscript?**

The scope of this article is limited to macromolecules. The application of similar methodologies to small molecules is beyond the scope of this paper and has been addressed in previous publications [1, 2, 3, 4, 5]. This clarification has been incorporated into the revised manuscript through a rewritten Introduction, as the focus was not clearly explained in the earlier version, see lines 64-66.

(6) *Zamora I, Fontaine F, Serra B, Plasencia G. High-throughput, computer assisted, specific MetID. A revolution for drug discovery. Drug Discov Today Technol. 2013 Spring;10(1):e199-205. doi: 10.1016/j.ddtec.2012.10.015. PMID: 24050248.*
(7) *Ahlqvist M, Leandersson C, Hayes MA, Zamora I, Thompson RA. Software-aided structural elucidation in drug discovery. Rapid Commun Mass Spectrom. 2015 Nov 15;29(21):2083-9. doi: 10.1002/rcm.7364. PMID: 26443410.*
(8) *Li AC, Chovan JP, Yu E, Zamora I. Update on hydrocodone metabolites in rats and dogs aided with a semi-automatic software for metabolite identification Mass-MetaSite. Xenobiotica. 2013 Apr;43(4):390-8. doi: 10.3109/00498254.2012.715697. Epub 2012 Aug 30. PMID: 22931213.*
(9) *Zelesky V, Schneider R, Janiszewski J, Zamora I, Ferguson J, Troutman M. Software automation tools for increased throughput metabolic soft-spot identification in early drug discovery. Bioanalysis. 2013 May;5(10):1165-79. doi: 10.4155/bio.13.89. PMID: 23721441.*
(10) *Bonn, B., Leandersson, C., Fontaine, F. and Zamora, I. (2010), Enhanced metabolite identification with MS^E^ and a semi-automated software for structural elucidation. Rapid Commun. Mass Spectrom., 24: 3127-3138. https://doi.org/10.1002/rcm.4753*

**(h) Line 107:111 – Datasets consisted of some linear and cyclic as well as natural and unnatural amino acids, and oligonucleotides, can represent the scope of application for analysis on a wide variety of compounds? How the coverage of applicability has been defined with compounds covered in the datasets. Please define**

The compounds selected for this study were chosen to represent a broad range of chemical diversity in terms of molecular weight (ranging from 700 to 7630 Da), structural features (including both linear and cyclic peptides), and composition (incorporating both natural and non-natural amino acids).

While it is not feasible for a single study to capture the full extent of chemical variability inherent to macromolecular therapeutics—given the vast and continuously expanding space of molecular designs—we have included representative cases that reflect common and relevant structural classes encountered in early drug discovery.

This has been clarified in the revised manuscript at lines 114-117 of the Introduction.

**(i) Line 141:144 – Repeated lines.**

This point has been addressed in the revised manuscript (see lines 155-157).

**3. Authors should provide an alternate approach when the fragmentation data does not match well with the theoretical.**

As described in the **Structure Visualization** subsection of *Materials and Methods*, the system supports two fragmentation strategies. In the non-expanded mode, the structure is represented at the monomer level, and fragmentation is modeled using classical ion types (a, b, c, x, y, z) following the Roepstorff–Fohlman nomenclature. In the expanded mode, the structure is visualized at the atomic level, and fragmentation is simulated by disconnecting all chemical bonds to generate theoretical fragments.

In cases where the fragmentation data does not align well with theoretical predictions, such discrepancies may arise from rearrangement reactions or secondary fragmentation pathways that are not currently being considered by our system. However, it is important to note that the atom/bond fragmentation strategy has been extensively validated over more than a decade in small-molecule studies (see attached references), during which no consistent evidence of missing rearrangement or secondary fragmentation mechanisms has been reported.

(1) *Zamora I, Fontaine F, Serra B, Plasencia G. High-throughput, computer assisted, specific MetID. A revolution for drug discovery. Drug Discov Today Technol. 2013 Spring;10(1):e199-205. doi: 10.1016/j.ddtec.2012.10.015. PMID: 24050248.*
(2) *Ahlqvist M, Leandersson C, Hayes MA, Zamora I, Thompson RA. Software-aided structural elucidation in drug discovery. Rapid Commun Mass Spectrom. 2015 Nov 15;29(21):2083-9. doi: 10.1002/rcm.7364. PMID: 26443410.*
(3) *Li AC, Chovan JP, Yu E, Zamora I. Update on hydrocodone metabolites in rats and dogs aided with a semi-automatic software for metabolite identification Mass-MetaSite. Xenobiotica. 2013 Apr;43(4):390-8. doi: 10.3109/00498254.2012.715697. Epub 2012 Aug 30. PMID: 22931213.*
(4) *Zelesky V, Schneider R, Janiszewski J, Zamora I, Ferguson J, Troutman M. Software automation tools for increased throughput metabolic soft-spot identification in early drug discovery. Bioanalysis. 2013 May;5(10):1165-79. doi: 10.4155/bio.13.89. PMID: 23721441.*
(5) *Bonn, B., Leandersson, C., Fontaine, F. and Zamora, I. (2010), Enhanced metabolite identification with MS^E^ and a semi-automated software for structural elucidation. Rapid Commun. Mass Spectrom., 24: 3127-3138. https://doi.org/10.1002/rcm.4753*

**4. How does this system handle data quality control and verification? Is it an entirely automated process? What threshold detail can it provide for the user to make an accurate decision?**

As described in section **Data Preprocessing** in the *Materials and Methods* and further discussed in response to point 16, the system uses an automated, multi-stage quality control and verification process for chromatographic peak detection and structure elucidation of metabolites.

The process begins with spectral noise analysis to eliminate low-intensity ions, followed by a blank comparison using Hodgkin and Pearson similarity indexes to remove background signals. Peaks are retained only if they are absent or significantly stronger than in the blank and are quantitatively assessed using the Negative Control Area Ratio.

To allow for flexibility across instrument types and analytical goals, the system provides user-configurable thresholds that influence the quality control process. Key configurable parameters include:

- Same peak tolerance (amu): Defines the acceptable mass error for identifying equivalent peaks; this is typically set according to the instrument's mass accuracy.
- Chromatogram automatic filtering threshold: Adjusts peak recognition sensitivity; higher values accept smaller peaks but risk merging noise, while lower values may exclude minor metabolites.
- MS automatic filtering threshold Controls MS spectrum filtering. Lower values increase processing speed but reduce sensitivity to low-abundance ions.
- MS/MS automatic filtering threshold: Tailors MS/MS noise reduction. Low thresholds yield cleaner spectra but risk missing relevant peaks; higher values may introduce noise that interferes with fragment matching.

Each detected peak is then scored based on isotope similarity, retention time, MS/MS data, and m/z accuracy. Structure elucidation uses both fragmentation of the parent molecule and from a predefined list of metabolic biotransformation reactions. A scoring system for reporting the potential structures candidates is made based on the matching, mismatching and metmatch peak intensities.

While the system automates much of the analysis, final verification is supported through expert review based on their data analysis criteria. The user is provided with retention times, MS areas, ppm error, MS/MS spectra, and classification of metabolites (e.g., first-generation or higher, unknown) to support expert-driven interpretation.

**5. These algorithms are based on fragmentation, isotope pattern, and m/z differences. Is there any possibility to combine these software with other data types for broad information, like 3D structure prediction? Also, quantitative details are required for more reliable weightage.**

These algorithms can indeed be combined with other data types to provide broader information, such as *in silico* prediction of peptide cleavage sites or peak selection using a machine learning model designed to replicate user-driven data analysis, thereby reducing the manual curation time required from experts in MetID. However, these aspects are beyond the scope of the current article, which focuses specifically on mass spectrometry data interpretation. Nonetheless, these directions are aligned with our ongoing research, and we are actively preparing manuscripts that address such integrative approaches.

**6. It would be great to mention the statistical analysis of the results throughout. Are these statistically significant? Also, mention these differences between the two algorithms.**

As requested, we have performed a statistical analysis to assess whether the differences in scores between the two algorithms (MaM and MiM) are statistically significant. A paired-samples *t*-test was conducted using the Score values reported in Tables 7, 8, and 9, excluding metabolites that were not found in one of the two approaches. The analysis yielded a *p*-value of 0.0002904, indicating that the difference in scores between MaM and MiM is statistically significant. This result has been added and highlighted in the Results section of the revised manuscript (see lines 654-656).

**7. For high score context, authors should mention a specific numerical threshold.**

We appreciate the reviewer’s suggestion. However, it is not feasible to define a numerical threshold for high-scoring structural proposals within our workflow. The score depends on the spectra quality of the experimental MS/MS spectra. For example, compounds lacking MS/MS fragmentation data will naturally yield low scores due to the absence of matching ions, whereas well-fragmenting compounds typically achieve higher scores because more fragment ions can be matched.

As described in the manuscript (see lines 326-329), the scoring system is inherently relative and context-dependent, as it is based on a combination of factors including the number and intensity of matched fragments, mismatches that help exclude unlikely structures, and the degree of spectral similarity to the parent compound.

The algorithm systematically generates hypothetical metabolite structures based on predefined biotransformations and applies a set of bond-breaking rules to simulate all possible fragment ions. Each proposed structure is scored according to how well these theoretical fragments match the observed MS/MS spectra. If multiple structures are compatible with the data, a Markush structural representation is automatically generated to represent this ambiguity. These structural assignments can be reviewed by an expert, who may accept one or more individual structures from the proposed Markush representation.

**8. Have previous studies conducted similar studies or comparisons? Please cite them and mention your novelty.**

Previous studies in metabolite identification (MetID), particularly for small molecules, have traditionally used peak detection algorithms based on the monoisotopic mass (MiM). This approach is widely adopted due to its suitability for compounds with relatively low molecular weights, where the monoisotopic peak is reliably detectable [1, 2].

However, to our knowledge, no prior studies have introduced or evaluated a peak detection algorithm based on the most abundant mass (MaM) specifically for macromolecular MetID. The novelty of our work lies in implementing and validating this MaM-based approach in an automated workflow, demonstrating its improved sensitivity and performance for large molecules where monoisotopic peaks are often low in intensity or undetectable.

In addition, we present two visualization strategies—expanded and non-expanded representations—that directly influence how biotransformations are computed. The non-expanded form, which operates at the monomer level, has not previously been applied in computational MetID workflows. Our comparison of expanded vs. non-expanded visualization illustrates the reduction in preprocessing time of the non-expanded algorithm.

We are not aware of any previous studies that combine both a MaM-based peak detection algorithm and dual structural visualization strategies in a fully automated macromolecular MetID workflow, making this contribution novel in both methodology and application.

(1) *Zamora I, Fontaine F, Serra B, Plasencia G. High-throughput, computer assisted, specific MetID. A revolution for drug discovery. Drug Discov Today Technol. 2013 Spring;10(1):e199-205. doi: 10.1016/j.ddtec.2012.10.015. PMID: 24050248.*
(2) *Bonn, B., Leandersson, C., Fontaine, F. and Zamora, I. (2010), Enhanced metabolite identification with MS^E^ and a semi-automated software for structural elucidation. Rapid Commun. Mass Spectrom., 24: 3127-3138. https://doi.org/10.1002/rcm.4753*

**9. MiM and MaM algorithms have the distinction of handling larger peptides. Elaborate underlying reason behind it. Different ways to process peptide size or mass could be a reason for it?**

As described in section **Structure Visualization (Peak detection (Monoisotopic Mass and Most Abundant Mass)** under *Data Preprocessing* in *Materials and Methods*, MiM relies on detecting the monoisotopic peak, which represents the molecular species composed entirely of the lightest isotopes (e.g., ¹²C, ¹⁴N). For small peptides, this peak is typically intense and reliably detected. However, as peptide size increases, the number of atoms increases, especially carbon atoms, which makes the probability of all atoms being the lightest isotopes extremely low. As a result, the monoisotopic peak becomes low in intensity or even absent, leading to reduced sensitivity for MiM in large molecules.

In contrast, MaM selects the most abundant isotope peak, regardless of whether it includes heavier isotopes like ¹³C. Since this peak remains intense even in larger peptides, MaM maintains higher sensitivity and is better suited for detecting and analyzing high-mass species.

**10. Please comment on how score differences reflect the practical reliability of the identification. Are the score differences substantial enough for metabolite identification? Also, mention scores for false-positive or false-negative identification. Authors should discuss the score threshold in all analysis.**

This point has already been addressed in our response to Point 7.

**11. Please comment: Is MiM less sensitive to certain types of enzyme degradation? OR the score differences related to the algorithm's sensitivity.**

The score differences between MiM and MaM are primarily related to the algorithmic sensitivity to molecular size. The algorithm calculates the monoisotopic mass using the lightest possible isotopes of each element in the molecule. This peak is typically the first in the isotope pattern and is most reliably detected in smaller molecules, where the chance of all atoms being the lightest isotopes (e.g., ¹²C, ¹⁴N) is relatively high.

However, as molecular size increases, so does the number of atoms in the molecule. With more atoms, especially carbon, the statistical probability that at least one heavier isotope (such as ¹³C) is present increases significantly. Each ¹³C adds approximately 1.003354 Dalton to the mass. This causes the most intense peak in the spectrum among all the isotope peaks of the molecule (used by the MaM algorithm) to appear at a higher m/z value than MiM.

Thus, for large molecules, the monoisotopic peak becomes low in intensity or may be undetectable, reducing the sensitivity of the MiM algorithm.

Regarding enzyme degradation, while it might be that certain proteases may have certain selectivity for peptide size and therefore affects in the sensitivity of the mass spectra measurement, investigating this relationship is beyond the scope of the current study and may be addressed in future work.

**12. Comment on how these algorithms handle false positives.**

The number of potential false positive chromatographic peaks (metabolites is primarily managed through user-configurable settings that adjust sensitivity thresholds. While the same parameter settings are applied in both the MiM and MaM algorithms, it will lead to different number of metabolites because the sensitivity of the mass spectral data depends on the size and the isotope used for the identification.

The system is designed to provide a number of solutions for expert review, allowing users to distinguish true metabolites from false positives based on their domain knowledge. To further reduce manual intervention of the user, our group is developing a complementary AI-driven approach aimed at automating peak selection, which will be detailed in future manuscript.

**13. You mention that the non-expanded structure visualization helped achieve the reduced processing time. Could you clarify why this approach is more efficient compared to the expanded visualization?**

As described in section **Structure Visualization (Expanded and Non-Expanded)** under *Data Preprocessing* in *Materials and Methods*, in the non-expanded structure visualization, the biotransformation is applied at the monomer level rather than at the atomic level. As a result, fewer chemical structures need to be constructed during the calculation process, which reduces processing time.

In contrast, the expanded visualization applies biotransformations at the atomic level, where all atoms and bonds are disconnected to generate fragments. This leads to a much larger number of fragments, resulting in higher computational demands and increased processing time.

To better illustrate this distinction, Table 10 has been added to the revised manuscript. It presents the processing times associated with different molecular weight ranges of peptides and the oligonucleotide examined in this study.

**14. Why were particular proteases like cathepsin G, neutrophil elastase, and trypsin chosen?**

This point has been addressed in the revised manuscript (see lines 125-131). The proteases and biological matrices used in the experimental incubations of these datasets represent key relevant proteolytic environments that therapeutic peptides are likely to encounter *in vivo*. This includes enzymes involved in gastrointestinal metabolism—where peptide hydrolysis primarily occurs—such as trypsin, chymotrypsin, elastase, and pepsin. The other proteases and matrices reflect metabolism in the liver, blood, and other physiological contexts, ensuring coverage of a broad range of relevant peptide degradation pathways.

**15. Line 247:251 – Generations of metabolites are classified with virtual screening based on predefined biotransformation reactions and m/z ratio, but differences among metabolites due to stereochemistry and reactions outside of the considered biotransformation limit the chances of correct analysis. How do authors believe that their method has overcome these limitations?**

Our method performs peak detection independently of whether a biotransformation is included in the predefined reaction list. As a result, metabolites formed through reactions not covered in the biotransformation reaction list can still be detected based on their spectral features. While a specific chemical structure may not be assigned in such cases, the presence of the metabolite will nonetheless be captured, allowing users to further investigate these findings.

**16. Line 411: 415 – classification of contaminations and background noise by unrecognised and irregular peaks may correspond to variation among metabolites of different generations. How do authors justify their parameters outlining the peak selection?**

As described in section **b) Automatic detection of the chromatographic peaks** under *Data Preprocessing* in the *Materials and Methods*, the chromatographic peak detection process begins with an initial spectral noise analysis. For each full scan, the noise level is estimated based on the change in slope between two consecutive shortlists of detected ions, and ions below this threshold are systematically eliminated.

To distinguish true peaks from background signals, a search is done to check whether the peak is also present in the blank sample. If the peak is detected in the blank, a peak alignment optimization is initiated using a combination of Hodgkin and Pearson similarity indexes computation, which compares both the chromatographic shape and peak intensity. A peak in the sample is excluded from further analysis if it exhibits a similar shape and equal or lower intensity compared to the corresponding peak in the blank.

This approach ensures that true peaks, including second-generation, higher-order, or unknown metabolites, are not discarded. These peaks are retained as long as they are not attributable to background noise or contamination. Furthermore, as detailed later in the manuscript, these retained peaks are classified (including also unknown as "red peaks") and evaluated with MS/MS data, isotope similarity, and retention time.

This peak selection works the same for small molecules and macromolecules, this strategy has been discussed in previous works of small molecules [1, 2, 3].

(1) *Zamora I, Fontaine F, Serra B, Plasencia G. High-throughput, computer assisted, specific MetID. A revolution for drug discovery. Drug Discov Today Technol. 2013 Spring;10(1):e199-205. doi: 10.1016/j.ddtec.2012.10.015. PMID: 24050248.*
(2) *Ahlqvist M, Leandersson C, Hayes MA, Zamora I, Thompson RA. Software-aided structural elucidation in drug discovery. Rapid Commun Mass Spectrom. 2015 Nov 15;29(21):2083-9. doi: 10.1002/rcm.7364. PMID: 26443410*
(3) *Zelesky V, Schneider R, Janiszewski J, Zamora I, Ferguson J, Troutman M. Software automation tools for increased throughput metabolic soft-spot identification in early drug discovery. Bioanalysis. 2013 May;5(10):1165-79. doi: 10.4155/bio.13.89. PMID: 23721441.*

**17. Line 315:317 – Dependency on the predefined databases should be countered if the structure is not matched?**

Our system can not rely on predefined metabolite databases for structure elucidation because the metabolites are formed on newly synthetized molecules and therefore, they can not exist in any database. Instead, metabolite structures are generated from the parent compound by applying a user-selectable list of predefined metabolic biotransformations reactions. So, when the structure is not matched, the system flags the metabolite structure as unknown, allowing users to review it manually or do further experimental validation.

**18. Could this approach be applied to high-throughput screening of peptide libraries or to analyse metabolites in clinical samples?**

This approach can indeed be applied to high-throughput screening of peptide libraries as well as to the analysis of metabolites in clinical samples. However, the exploration and discussion of such sample types are beyond the scope of the current article.
